# First draft genome for the sand-hopper *Trinorchestia longiramus*

**DOI:** 10.1038/s41597-020-0424-8

**Published:** 2020-03-09

**Authors:** Ajit Kumar Patra, Oksung Chung, Ji Yong Yoo, Min Seop Kim, Moon Geun Yoon, Jeong-Hyeon Choi, Youngik Yang

**Affiliations:** 1 Ewha Womans University, Seoul, 03760 South Korea; 2 Clinomics Inc., Ulsan, 44919 South Korea; 3 National Marine Biodiversity Institute of Korea, Seocheon, 33662 South Korea

**Keywords:** Phylogenetics, DNA sequencing, Evolutionary ecology, Sequence annotation, Genome

## Abstract

Crustacean amphipods are important trophic links between primary producers and higher consumers. Although most amphipods occur in or around aquatic environments, the family Talitridae is the only family found in terrestrial and semi-terrestrial habitats. The sand-hopper *Trinorchestia longiramus* is a talitrid species often found in the sandy beaches of South Korea. In this study, we present the first draft genome assembly and annotation of this species. We generated ~380.3 Gb of sequencing data assembled in a 0.89 Gb draft genome. Annotation analysis estimated 26,080 protein-coding genes, with 89.9% genome completeness. Comparison with other amphipods showed that *T*. *longiramus* has 327 unique orthologous gene clusters, many of which are expanded gene families responsible for cellular transport of toxic substances, homeostatic processes, and ionic and osmotic stress tolerance. This first talitrid genome will be useful for further understanding the mechanisms of adaptation in terrestrial environments, the effects of heavy metal toxicity, as well as for studies of comparative genomic variation across amphipods.

## Abstract (summary)

**Table:** 

Measurement(s)	DNA • RNA • sequence_assembly • sequence feature annotation
Technology Type(s)	DNA sequencing • RNA sequencing • sequence assembly process • sequence annotation
Sample Characteristic - Organism	Trinorchestia longiramus

Machine-accessible metadata file describing the reported data: 10.6084/m9.figshare.11897430

## Background & Summary

Amphipoda is an order of malacostracan crustaceans, composed of more than 228 families with over 10,200 species^1^. Most members of Amphipoda are found in aquatic environments, with both freshwater and marine species that occur in diverse habitats^2–6^. However, only a few amphipods in the family Talitridae are found in terrestrial regions close to the water, and others are “semi-terrestrial,” with both littoral and terrestrial representatives^7^.

Talitrids are one of the prevailing macrofaunal groups in coastal regions that live along the interface between the water and land. The coastal talitrids, also known as “sand-hoppers,” are considered key species for energy flow to higher trophic levels^8^. They play a crucial role in food web dynamics by feeding on algal-biomass^9^ and detritus along sandy beaches. They then become the source of food for many invertebrates, fish, and birds^4,8^. Unfortunately, anthropogenic activity contributes to various types of pollutants in the coastal ecosystem, which impacts the survival of talitrids^10–12^ and other macrofauna^13–15^. For this reason, many talitrids are used as model organisms for studies of metal toxicity^10–12^. In addition, previous work on talitrids examined levels of genetic variation^16,17^, behavioral adaptations^18^, osmoregulation^19^, and orientation studies^20^. Most of these studies were carried out along the North Sea and the Mediterranean Sea regions.

Despite such biological and ecological significance, no genome studies have been performed on any talitrid species, and only three genomes have been studied among the entire amphipod order. These included (1) *Eulimnogammarus verrucosus* (Family: Eulimnogammaridae)^21^, a freshwater amphipod from Baikal Lake; (2) *Hyalella azteca* (Family: Hyalellidae)^22^, another freshwater amphipod that lives by burrowing in the sediments; and (3) *Parhyale hawaiensis* (Family: Hyalidae)^23^. *Trinorchestia longiramus* Jo, 1988^24^ is in the family Talitridae and is highly abundant in sandy beaches of South Korea^24–26^ and Japan^27^. Because of its widespread range, simplicity to rear in the laboratory, and relatively small genome size, *T*. *longiramus* can be a useful model organism for developmental biology, ecology, evolution, and studies of metal bioaccumulation.

In this study, we present the first draft genome of *T*. *longiramus* using high-throughput sequencing. We isolated genomic DNA from whole tissues, constructed two paired-end (PE) and four mate pair (MP) libraries, which were then sequenced with the Illumina HiSeq. 2500 platform. The estimated genome size of *T*. *longiramus* is ~1.116 Gb. The draft genome was assembled into 30,897 scaffolds (N50 = 120.57 kb), with a total size of 0.89 Gb, which corresponds to approximately 79.43% of the estimated genome size. Structural annotation of the genome yielded 26,080 genes. BUSCO analysis revealed gene space completeness of 89.9%. Of the total genes predicted, 14,959 genes were functionally annotated with InterProScan^28^. The lineage containing *T*. *longiramus* reveals gene expansion of particular gene families, including those related to response to stress, homeostatic process, transmembrane transport, and signal transduction. A phylogenetic analysis with related amphipod and arthropod species suggests that *T*. *longiramus* diverged from the *H*. *azteca* during the Late Cenozoic era. This first talitrid genome will be useful for further understanding the mechanisms of adaptation in terrestrial environments, the effects of heavy metal toxicity, as well as for studies of comparative genomic variation across amphipods.

## Methods

### Sample collection and extraction of DNA and RNA

*T*. *longiramus* samples were collected from the coast (37°41′29″N, 129°2′2.7″E) of South Korea. They were captured by hand from exposed and sheltered sandy beaches. Samples were preserved immediately in 95% ethanol for genome sequencing and stored in liquid nitrogen for RNA extraction.

DNA was extracted from a pool of seven individuals using a conventional phenol-chloroform protocol^29^. The purified DNA was resuspended in Tris-EDTA (TE) buffer (TE; 10 mM Tris–HCl, 1 mM EDTA, pH 7.5). For RNA isolation, several frozen whole bodies were mortar-pulverized in liquid nitrogen. The purified RNA was extracted in lysis buffer, containing 35 mM EDTA, 0.7 M LiCl, 7.0% SDS, and 200 mM Tris–Cl (pH 9.0), following the protocol by Woo *et al*.^30^. The purified RNA was eluted in DEPC-treated water and stored at −20 °C.

### Short and long DNA fragment library construction

Two PE libraries were prepared with insert size 350 bp using the TruSeq DNA Sample Prep kit (Illumina). In addition, four MP libraries were prepared with insert sizes 3, 5, 8, and 10 kb using the Nextera Mate Pair Sample Preparation kit (Illumina). All libraries were sequenced on an Illumina HiSeq. 2500 instrument, with 251 bp reads for the PE libraries and 101 bp reads for the MP libraries. We generated a total of 592,854,944 (149 Gbp) PE reads and 2,291,660,676 (231 Gbp) MP reads (Table 1).

**Table 1:** Sequence libraries and data yield from Illumina DNA and RNA sequencing.

	Library type	Insert Size (bp)	Read Length (bp)	Raw bases (Gb)	Raw reads	SRA accessions
DNA	Paired-end (PE)	350	251	37.616	149,863,175	SRR9098167
		350	251	37.616	149,863,175	SRR9098167
		350	251	36.788	146,564,297	SRR9098168
		350	251	36.788	146,564,297	SRR9098168
	Total			148.808	592,854,944	
	Mate-pair (MP)	3 K	101	28.942	286,552,798	SRR9098169
		3 K	101	28.942	286,552,798	SRR9098169
		5 K	101	29.710	294,156,030	SRR9098170
		5 K	101	29.710	294,156,030	SRR9098170
		8 K	101	27.904	276,279,897	SRR9098171
		8 K	101	27.904	276,279,897	SRR9098171
		10 K	101	29.173	288,841,613	SRR9098172
		10 K	101	29.173	288,841,613	SRR9098172
	Total			231.458	2,291,660,676	
RNA	PE	140	101	6.204	61,429,733	SRR9112990
		140	101	6.204	61,429,733	SRR9112990
	Total			12.408	122,859,466	

### RNA short fragment and PacBio Iso-seq sequencing

For short fragment sequencing, a PE library was prepared with the Truseq mRNA Prep kit (Illumina) from total mRNA, which was subsequently sequenced on an Illumina Hiseq. 2500 with read lengths of 101 bp (Table 1). A total of 122,859,466 (12 Gbp) PE reads were sequenced.

For PacBio Iso-Seq sequencing, three sequencing libraries (1–2, 2–3, and 3–6 kb) were prepared from polyA+ RNA according to the PacBio ISO-sequencing protocol. A total of six Single-Molecule Real-Time cells were run on a PacBio RS II system by DNALink Co. From a total of 350,860 reads, 72,517 high-quality transcripts were generated (Table 2).

**Table 2:** Sequencing libraries and data yields from PacBio RNA sequencing.

Library size (Kb)	Average read Length (bp)	Raw bases (Gb)	Raw reads	Polished high-quality isoforms	SRA accession
1–2	1,238	0.027	21,522	72,517	SRR9112991
	2,070	0.219	105,671		
2–3	2,209	0.070	31,546		
	2,522	0.251	99,339		
3–6	2,810	0.029	10,278		
	3,656	0.302	82,504		
Total	2,418	0.896	350,860		

### k-mer distribution and genome size estimation

**Fig. 1:**
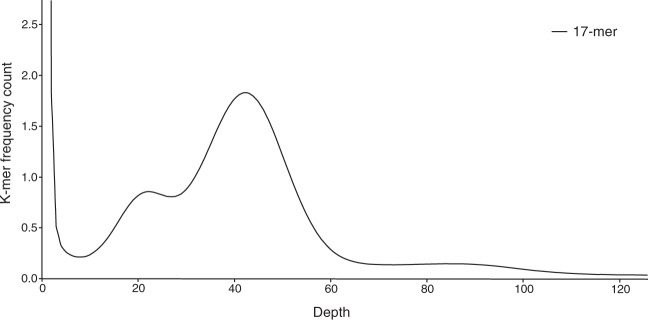
Genome size estimation by k-mer distribution.

Prior to estimating the genomic size, we processed raw reads as follows. We discarded low-quality (<Q20) PE reads and those that contained the Truseq index and universal adapters. We then merged the high-quality PE reads using FLASH^31^, with default options to avoid double counting of overlapping reads. The estimated genome size of *T*. *longiramus* was ~1.116 Gb based on a k-mer distribution (K = 17) analysis run with JELLYFISH^32^. The main peak exists at k-mer depth 42, which was used for genome size estimation (Fig. 1).

### Genome assembly

**Fig. 2:**
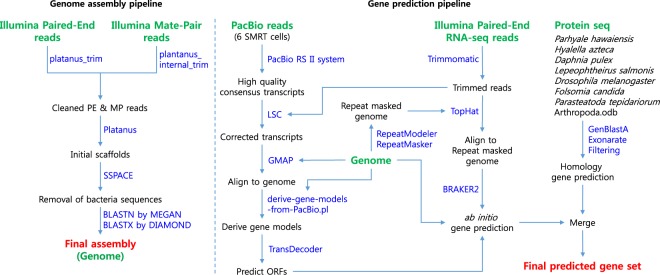
*T*. *longiramus* genome assembly and gene prediction workflow.

Assembly, adapters, low-quality reads, and uncalled bases were trimmed from PE and MP raw reads using Platanus_trim and Plantanus_internal_trim, respectively. Initial assembly was performed with Platanus^33^ based on automatically optimized multiple k-mer values. We executed individual commands “assemble,” “scaffold,” and “gap_close” in the Platanus assembler suite, successively. For the “assemble” stage, we assigned the maximum memory usages as 2,048 G, but all the other stages were executed with default options. Scaffolds larger than 1,000 bp in length scaffolded using trimmed PE and MP reads in SSPACE^34^ (Fig. 2). Finally, we filtered out two bacterial sequences with more than 500 BLASTN bit scores of 90% alignment coverage identified in MEGAN^35^. We re-confirmed using BLASTX with a non-redundant database in DIAMOND^36^. Table 3 shows the assembly statistics for Platanus, SSPACE, and the final assembly.

**Table 3:** Statistics of the *T. longiramus* genome assembly.

	Platanus	SSPACE	Final
Scaffolds	1,025,695	30,899	30,897
Scaffolds (>1000)	63,362	30,899	30,897
Total Length	1,022,727,337	886,386,416	886,359,443
Total Length (>1000)	828,517,177	886,386,416	886,359,443
Maximum length	1,019,543	1,680,077	1,680,077
N50	74,013	120,570	120,570
Gap	16,045,251	73,899,800	73,869,646

### Repeat annotation

To annotate repetitive elements, we first identified tandem repeats using the Tandem Repeats Finder^37^. Transposable elements (TEs) were identified by combining *de novo* (RepeatModeler)^38^ and homology-based approaches (Repbase^39^, RepeatMasker^40^, and RMBlast^40^). TEs accounted for 20.35% of the genome, with tandem repeats accounting for the largest portion (6.18%) (Table 4).

**Table 4:** Statistics of repetitive elements.

	Total (bp)	% of genome
DNA	45,354,677	5.12
LINE	23,869,606	2.70
LTR	11,269,516	1.27
Low_complexity	1,202,626	0.14
SINE	163,811	0.02
Satellite	308,670	0.03
Simple_repeat	10,854,020	1.22
TandemRepeat	54,776,419	6.18
Unknown	48,880,228	5.51
Unspecified	397,465	0.04
Total	180,352,209	20.35

### Gene prediction and annotation

The protein-coding genes were predicted by combining *ab initio* and homology-based gene prediction methods (Fig. 2). For the *ab initio* gene prediction, BRAKER^41^ predicted 67,698 genes, which incorporated outputs from GeneMark-ET^42^ and AUGUSTUS^43^. GeneMark-ET predicts genes with unsupervised training, whereas AUGUSTUS predicts genes with supervised training based on intron and protein hints. We generated two hint files from an Illumina RNA-seq and PacBio ISO-seq. Tophat^44^ was used to align RNA-seq reads to the repeat-masked genome assembly. We proceeded with Iso-seq to obtain the protein sequences, as described in Minoche *et al*.^45^: (1) run LSC^46^ to correct errors for full-length transcripts, (2) align the corrected transcripts to the genome using GMAP^47^, and (3) generate gene models from aligned sequences and extract the protein sequence from the generated gene model using Transdecoder^48^. We obtained 1,573 protein sequences, which were used to generate protein hints for AUGUSTUS by running Exonerate^49^. To remove incomplete gene sequences from genes predicted by BRAKER, we filtered out the predicted coding sequences (CDSs) using the following two criteria: 1) CDSs that contained premature stop codons and (2) CDSs that were not supported by hints. Finally, a total of 23,985 protein-coding genes were estimated by *ab initio* prediction (Table 5).

**Table 5:** Statistics of predicted protein-coding genes.

	Number	Average transcript length (bp)	Average CDS length (bp)	Average intron length (bp)
*De novo*	23,985	8,060.4	242.1	1,616.3
Homology	9,913	7,836.5	200.3	1,744.8
Merged	26,080	7,720.7	242.9	1,744.8

For the homology gene predictions, we searched the assembly of T. longiramus against Daphnia pulex, Drosophila melanogaster, Folsomia candida, H. azteca, Lepeophtheirus salmonis, Parasteatoda tepidariorum, P. hawaiensis, and arthropoda in orthoDB using TBLASTN^50^ with an E-value cutoff of 1E-5. Matching sequences were clustered using GenBlastA^51^, and only best-matched regions were retained. Then, gene models were predicted using Exonerate^49^. Predicted gene sequences that did not meet the above criteria were discarded. As a result, a total of 9,913 genes were predicted by a homology-based approach (Table 5).

Finally, we combined the two outputs by placing homology predictions to *ab initio* prediction only when there is no conflict. As a result, 26,080 protein-coding genes were predicted for the *T*. *longiramus* draft genome (Table 5). Gene Ontology for the predicted genes were annotated using InterProScan with various databases^52^, including Hamap^53^, Pfam^54^, PIRSF^55^, PRINTS^56^, ProDom^57^, PROSITE^58^, SUPERFAMILY^59^, and TIGRFAM^60^ (Gene Ontology annotation of *T*. *longiramus*)^61^.

## Data Records

All DNA and RNA raw reads have been deposited in the NCBI SRA (Table 1) under the SRA study accession SRP199018^62^. The whole genome shotgun sequencing project was deposited in GenBank under accession VCRD01000000^63^. In addition, the assembled genome was submitted to NCBI Assembly and is available with accession no. GCA_006783055.1^64^. Gene Ontology annotation table has been deposited to Figshare^61^ 10.6084/m9.figshare.8217854.

## Technical Validation

### DNA and RNA sample quality

DNA quality was assessed using Nanodrop, 1% agarose gels, Qubit fluorometer, and the Qubit HS DNA assay reagents. The RNA integrity was assessed using Nanodrop and an Agilent 2100 Bioanalyzer electrophoresis system (Agilent, Santa Clara, CA, USA).

### Illumina libraries

Ready-to-sequence Illumina libraries were quantified by qPCR using the SYBR Green PCR Master Mix (Applied Biosystems), and library profiles were evaluated with an Agilent 2100 Bioanalyzer (Agilent Technologies, Santa Clara, CA, USA).

### Genome assembly and gene prediction quality assessment

The length statistics of the genome assembly were assessed by QUAST^65^. The total assembly length is 0.89 Gb, which corresponds to 79.43% of the estimated genome size. The final scaffold N50 is 120.57 kb (Table 3). Genome completeness was evaluated using BUSCO^66^, with Arthropoda conserved genes databases. The genome assembly, after removing bacteria sequences from SSPACE, revealed a complete BUSCO value of 88.3%. However, in predicted genes, BUSCO completeness was higher (89.9%) (Table 6).

**Table 6:** BUSCO assessment of genome assembly and gene prediction.

Genome assembly	# Scaffolds	BUSCO (Arthropoda)
Platanus	63,362	C:86.0%[S:84.3%,D:1.7%],F:6.3%,M:7.7%,n:1066
SSPACE	30,899	C:88.3%[S:86.8%,D:1.5%],F:4.5%,M:7.2%,n:1066
Final	30,897	C:88.3%[S:86.8%,D:1.5%],F:4.5%,M:7.2%,n:1066
**Gene prediction**	**# Genes**	
Final	26,080	C:89.9%[S:85.3%,D:4.6%],F:6.6%,M:3.5%,n:1066

### Comparison with other arthropod genomes

We performed an extensive comparison of orthologous genes among 12 arthropod genomes (*Trinorchestia longiramus*, *Daphnia pulex*, *Drosophila melanogaster*, *Folsomia candida*, *H*. *azteca*, *Lepeophtheirus salmonis*, *Parasteatoda tepidariorum*, *P*. *hawaiensis*, *Oithona nana*, *Eulimnadia texana*, *Strigamia maritima*, and *Tigriopus kingsejongensis*) using OrthoMCL^67^.

**Fig. 3:**
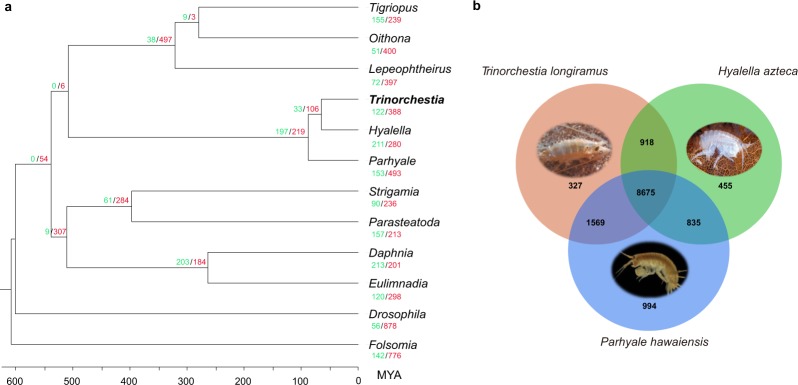
Comparison of orthologous genes. (**a**) Gene family expansion and contraction in arthropod species. Numbers designate the gene families that have expanded (green) and contracted (red) after the split from the common ancestor. Divergence time is scaled in millions of years. (**b**) A Venn diagram of unique and shared orthologous gene clusters in *T*. *longiramus*, *P*. *hawaiensis*, and *H*. *azteca*.

After orthologous gene clustering, 490 single-copy protein sequences were aligned using MUSCLE^68^. Low alignment quality regions were filtered using trimAl^69^. A phylogenetic tree was constructed using RAxML^70^, with the PROTGAMMAJTT model (100 bootstrap replicates). Divergence time was calculated using MEGA7^71^ with the Jones–Taylor–Thornton model and the previously determined topology (Fig. 3a). Calibration times of *Parasteatoda*–*Drosophila* divergence (601 MYA) and *Strigamia*–*Drosophila* divergence (583 MYA) were taken from the TimeTree database^72^. We found that *T*. *longiramus* diverged from *H*. *azteca* during the Early Cenozoic era, approximately 55 million years ago.

A gene expansion and contraction analysis was conducted using the CAFE program^73^ with the estimated phylogenetic information. A total of 122 gene families have expanded, and 388 gene families were contracted in *T*. *longiramus*. Fisher’s exact test (p-value ≤ 0.05) was used to identify functionally enriched categories among expanded genes relative to the “genome background,” as annotated by Pfam (Supplementary Table 1). We observed that gene families associated with transferring glycosyl and acyl groups, ATPase activity, response to stress, homeostatic process, and transmembrane transport have expanded. Among transmembrane transport activities, we found that sodium/hydrogen exchanger genes were responsible for a wide range of cellular functions, such as cation movement, homeostasis, regulation of pH, and tolerating ionic and osmotic stress^74^. We also found several genes, such as ABC transporters responsible for efflux toxicants out of the cells^75^, sodium-independent organic anion transporter required for uptake of organic amphipathic compounds, and xenobiotic drugs^76^.

A Venn diagram of orthologous gene clusters was drawn on the basis of the protein sequences from *T*. *longiramus* (26,080 proteins) and two amphipods: *H*. *azteca* (17,509 proteins) and *P*. *hawaiensis* (28,617 proteins) (Fig. 3b). *T*. *longiramus* has 327 unique orthologous gene clusters found among these three genomes. Among these unique gene clusters, the top three gene clusters are DNA- and RNA-mediated transposition, iron ion binding, and DNA metabolic process. Several unique genes also were found in expanded gene families mentioned above (Supplementary Table 1).

## Usage Notes

All analyses were conducted on Linux systems, and optimal parameters are given in the Code availability section.

**Table 7:** A list of software and parameters used for genome analysis.

Softwares	Version	Parameters/Commands
FLASH	1.2.11	default
JELLYFISH	2.2.6	-C -m 17
Platanus trim	1.0.7	platanus_trim (for PE reads), platanus_internal_trim (for MP reads)
Platanus	1.2.4	step-1: assemble -m 2048, step-2: scaffold, step-3: gap_close
SSPACE Standard	3.0	default
DIAMOND	0.9.24	default
MEGAN	6.15.2	default
QUAST	4.5	default
BUSCO	3.0.2	-l arthropoda_odb9
RepeatMasker	4.0.7	-e ncbi -pa 4
RepeatModeler	1.0.10	-engine ncbi -pa 4
LSC	2.0	default
GMAP	2018-07-04	-B 5
derive-gene-models-from-PacBio.pl		default
TransDecoder	3.0.1	step-1: TransDecoder.LongOrfs, step-2: TransDecoder.Predict
Tophat	2.1.1	–microexon-search–mate-std-dev 26–mate-inner-dist 38–min-intron-length 30–min-coverage-intron 30–min-segment-intron 30
GenBlastA	1.0.4	-p T -e 1e-5 -g T -f F -a 0.5 -d 100000 -r 100 -c 0.01 -s -100
Exonerate	2.2.0	–model protein2genome –percent 30 –showvulgar no –showalignment yes–showquerygff no –showtargetgff yes –targetchunkid 1–targetchunktotal 100
BRAKER	2.0	–species = *T. longiramus* – AUGUSTUS_CONFIG_PATH = augustus/config – AUGUSTUS_BIN_PATH = augustus/bin – AUGUSTUS_SCRIPTS_PATH = augustus/scripts – GENEMARK_PATH = gm_et/gmes_petap – bam = tophat/accepted_hits.bam–prot_seq = PacBio-derived.gene-models.transdecoder.pep.fasta –alternatives-from-evidence = true –prg = exonerate
InterProscan	5.16–55.0	-appl HAMAP,ProDom,PRINTS,Pfam,TIGRFAM,SUPERFAMILY,ProSitePatterns,ProSiteProfiles -goterms -iprlookup
OrthoMCL	2.0.9	-I 1.5
MUSCLE	3.8.31	default
ETE	3.1.1	trimal -gappyout
RAxML	8.2.10	-m PROTGAMMAJTT
MEGA	7.00	megacc
CAFE	4.0	default

## Supplementary information

Supplementary Table 1

## Data Availability

The software versions, settings, and parameters are described in Table 7. If not mentioned otherwise, the command line at each step was executed using default settings.
